# Training the equine respiratory muscles: Ultrasonographic measurement of muscle size

**DOI:** 10.1111/evj.13598

**Published:** 2022-06-19

**Authors:** Laura E. Fitzharris, Melanie J. Hezzell, Alison K. McConnell, Kate J. Allen

**Affiliations:** ^1^ Bristol Veterinary School University of Bristol Bristol UK; ^2^ Independent Consultant Bournemouth UK

**Keywords:** horse, inspiratory muscle training, thickness, ultrasound

## Abstract

**Background:**

Limited information exists regarding changes in the size of respiratory and locomotor muscles in response to exercise training in the Thoroughbred racehorse.

**Objectives:**

To describe and compare the responses of the respiratory and locomotor muscles to conventional exercise training and inspiratory muscle training (IMT).

**Study design:**

Prospective randomised controlled trial.

**Methods:**

Thoroughbred racehorses, in training for competition in National Hunt races, were recruited from two training establishments. Ultrasonographic images were obtained for selected muscles of the upper airway, diaphragm, accessory respiratory, and locomotor systems and their sizes measured. Examinations were performed at three timepoints: (A) when unfit, (B) following 12 weeks of conventional exercise training and (C) following 10–12 weeks continued training at race fitness. In addition, horses at yard 1 performed IMT, between timepoint B and C, and were randomly assigned into high‐load (treatment) or low‐load (control) group. Repeated measures models were constructed to compare the change in muscle measurements over time, and to investigate the effects of yard, previous airway surgery and IMT on the change in ultrasonographic size measurements obtained.

**Results:**

Upper airway muscle size increased in response to conventional race training between timepoints A–C, and B–C. Diaphragm size increased in response to conventional exercise training between timepoints A and B. The diaphragm size of horses that undertook high‐load IMT was either maintained or increased, whereas diaphragm size decreased in horses that undertook low‐load IMT or no IMT between timepoints B and C. A significant interaction between gluteal muscle size and airway surgery status was observed, with greater gluteal muscle thicknesses measured in horses that had not previously undergone airway surgery (left gluteal 3.9%, *p* < *0.001*; right 4.5%, *p* = *0.04*).

**Main limitations:**

Low number of horses underwent IMT.

**Conclusions:**

Respiratory and locomotor muscles increase in size in response to conventional exercise training, with a further change in diaphragm size in response to inspiratory muscle training.

## 1 INTRODUCTION

The demands of the respiratory system increase substantially during exercise, and this system is thought to be the limiting factor which determines athletic performance.^1^ During exercise there is a significant increase in respiratory frequency, tidal volume and minute ventilation.^1^ Despite these responses, racehorses develop hypoxaemia, oxygen desaturation and hypercapnia during strenuous exercise.^1^, ^2^ Although substantial increases in VO_2max_ occur with training,^1^ these occur mainly through cardiovascular and muscle adaptation; the structural and functional properties of the lungs and airways do not change in response to training.^3^ As such, the respiratory muscles may prove to be the key component of the respiratory system which can adapt to a training stimulus and are the subject of this investigation. The diaphragm is the primary respiratory muscle^4^ and plays an important role during exercise. Studies conducted in human athletes have identified diaphragm fatigue as a key factor in athletic performance limitation via a respiratory muscle fatigue‐induced metaboreflex.^5^ It is currently unknown whether this exercise‐induced metaboreflex occurs in the horse and whether diaphragm fatigue plays a role in limiting equine performance.

Investigations conducted in human athletes have shown an increase in skeletal muscle mass in response to exercise training,^6^ with an increase in the thickness of the diaphragm in response to global strength training^7^ and specific inspiratory muscle training (IMT).^8^ Inspiratory muscle training applies a training stimulus to the inspiratory muscles in isolation of whole‐body training. Investigations in human athletes have shown IMT can delay the onset of inspiratory muscle fatigue^9^ which can lead to improvements in athletic performance.^10^ Recent studies have established the successful application of IMT, performed at rest, in Thoroughbred racehorses, demonstrating an increase in inspiratory muscle strength and change in upper airway muscle function following a period of IMT.^11^, ^12^, ^13^ Ultrasonographic measurement is a non‐invasive and repeatable method to assess muscle thickness and cross‐sectional area, and is performed in human athletes to evaluate the effect of training and to measure the response to rehabilitation.^14^, ^15^, ^16^ Previous investigations in racehorses have demonstrated a significant association between ultrasonographic measurement of left ventricular dimensions, and fat free mass and published performance ratings.^17^, ^18^ Further assessment of the relationship between physiologic variables, such as diaphragm thickness, and race performance are of high interest. To the authors' knowledge, there are no studies that have measured the gross change in muscle size, measured non‐invasively using ultrasonography, in Thoroughbred racehorses in response to conventional race training or IMT.

The aim of this investigation was to measure the size of selected respiratory and locomotor muscles in Thoroughbred racehorses at three timepoints during the race season to investigate any changes in response to conventional exercise training for National Hunt racing and IMT. It was hypothesised that there would be an increase in respiratory and locomotor muscle size with exercise training, with a further increase in respiratory muscle size with IMT. A secondary aim was to assess whether there was an association between ultrasonographic measurements of muscle thickness and assigned performance ratings at the time of assessment.

## 2 MATERIALS AND METHODS

### 2.1 Population

Thoroughbred racehorses, in training for National Hunt racing, were recruited from two separate training establishments. Recruitment criteria required horses to be considered ‘unfit’ but healthy and not to have undergone any routine exercise for a minimum of 8 weeks before the first examination. All horses were to enter a conventional exercise programme to train for competition in National Hunt races. Sample size calculations were performed using GLIMMPSE software (Version 2, https://v2.glimmpse.samplesizeshop.org/#/) based on preliminary data^19^ estimating a 10% change in muscle size in response to a training stimulus with a 15% drop out rate. Based on this information, with power *β* = 0.8 and type I error *α* = 0.05, we aimed to recruit a minimum of 44 horses for each part.

### 2.2 Horse information

Information obtained from each yard, for every horse recruited, included details of the horse's fitness level at each timepoint (subjectively determined by the trainer) and whether the horse had undergone airway surgery. Additional information about training methods and regimes were obtained at the end of the study period and was acquired verbally via the telephone and followed the same transcript for both yards.

### 2.3 Examination

All examinations took place in the horse's stable and were performed without sedation, with the horse restrained using a headcollar and lead rope only. The horses were examined on three occasions, firstly at timepoint A (July) when considered ‘unfit’, at timepoint B (October) when considered ‘race fit’ following 12 weeks of exercise training, and at timepoint C (January) following continued race training at race fitness for 10 weeks at yard 1 and 12 weeks at yard 2.

### 2.4 Exercise training

Each horse undertook an individually tailored exercise programme that was determined by each trainer. The overall aim was to increase fitness from unfit at timepoint A to race fitness at timepoint B and maintain race fitness to timepoint C.

### 2.5 Inspiratory muscle training

Following examination at timepoint B, the horses at yard 1 commenced an IMT programme. The horses were randomly assigned, using a random number generator, into either a high‐load (treatment) group or low‐load (control) group. The IMT was applied using a bespoke mask and inspiratory pressure threshold valves.^6^ The high‐load group followed an IMT protocol that gradually increased the inspiratory pressure applied every 4 days (5, 10, 12.5, 15, 20 cmH_2_O using Intersurgical® [C‐PEEP™] valves then in increments of 2.5 cmH_2_O using the second POWERbreathe® valve, Medic Classic, POWERbreathe International Ltd.) (Table S1). The low‐load group underwent sham treatment with a low training load of 2.5 cmH_2_O. In addition to this, the low‐load horses took an extra five breaths of a greater load every 5 days, which gradually increased up to a maximum of 20 cmH_2_O (5, 10, 12.5, 15, 20 cmH_2_O) (Table S1) to acclimatise the horse to opening a valve with a higher pressure without a having training effect on the muscles. For both groups, the IMT involved two sessions of 3 min duration performed back‐to‐back with a short break in between, undertaken 5 days per week over a period of 10 weeks. A minimum of 40 IMT sessions were to be completed before repeat examination. Horses did not undertake IMT for 48 h prior to racing. The IMT was performed by either the primary author (LF) or by a research assistant, who had undergone specific training on how to use and apply the IMT correctly.

### 2.6 Ultrasonographic examination

#### 2.6.1 Equipment

Ultrasonographic examinations were performed by a single observer (LF) using a portable ultrasound machine (GE LOGIQ™ e R7; GE Healthcare). A linear transducer (6–13 MHz) with a 43 mm footprint was used for all examinations except the upper airway muscles, where a 25 mm footprint ‘hockey stick’ transducer (8–18 MHz) was used. Standardised settings for each muscle were used based on the preliminary investigations,^16^ (Methods S1) to provide optimal images.

#### 2.6.2 Technique

Ultrasonographic examination was performed using standardised protocols^16^ (Methods S1), with the horses standing square and still, undertaking even breathing at a rate between 6 and 14 breaths per minute (brpm). Images of the following muscles and structures were obtained: cricothyroideus (CT), thyrohyoideus (TH), geniohyoideus (GH), genioglossus (GG), basihyoid bone depth at the base of the lingual process, sternothyrohyoideus (STH), diaphragm, extensor carpi radialis (ECR), gluteus medius (GM) and vastus lateralis (VL). All structures were examined bilaterally where appropriate.

During ultrasonography, contact was achieved by dampening the skin with isopropyl alcohol applied with a gauze swab. For static images, a total of five images was obtained for each muscle. For dynamic images of the diaphragm, a total of two cineloops, each of 30 s duration, were obtained. Between obtaining each static image or cineloop the probe was removed from the horse, and additional isopropyl alcohol applied before the next image or cineloop was captured. Static images and cineloops were rejected at the time of acquisition if the horse undertook a spontaneous deep breath, had an elevated (>14 brpm) or reduced (<6 brpm) respiratory rate, or moved during image acquisition.

#### 2.6.3 Measurements

Measurements were performed off‐line at a later date. Standardised protocols^19^, ^20^ (Methods S1) were used to measure each image. Briefly, for each static image the muscle depth was measured once on each image at a specified location. For the cineloops, each cineloop was reviewed frame‐by‐frame, and static images at peak inspiration and peak expiration were obtained and saved for measurement. A minimum of three inspiration and three expiration images were obtained for each cineloop. Measurements were performed on the ultrasound machine using the electronic callipers.

The term ‘size’ is used in this manuscript to refer to the measurements obtained. For most muscles, the thickness was measured, for two muscles the cross‐sectional area was measured (CT and STH), and the depth of the basihyoid bone was measured.

### 2.7 Athletic performance

All horses examined at timepoints B and C were included in the analysis. Additional horses, deemed to be at race fitness, were recruited from Yard 1 at timepoint C. Information recorded for each horse at each timepoint included: weight, yard, airway surgery status (yes or no) and number of seasons in training. The ultrasonographic size measurements of the diaphragm on the left side during inspiration and left gluteal muscle thickness were recorded for each horse at each timepoint. In addition, rump fat thickness measurements were measured on the images obtained for gluteal muscle assessment for all horses examined at timepoint C. Where applicable, for the horses examined at Yard 1, the index of inspiratory muscle strength (timepoints B & C) (L. Fitzharris et al., unpublished data) and inspiratory muscle training group (high‐load or low‐load at timepoint C) were recorded. Data extracted for analysis of performance parameters were accessed from an online database (https://www.racingpost.com). For each horse, the official rating (OR) at the time of each examination was recorded.

### 2.8 Data analysis

Data were recorded in Microsoft Excel® and analysis performed using SPSS® (version 24). The data were assessed for normality by visual inspection of histogram plots and a Shapiro–Wilk test. Repeated measures models were constructed to compare the change in muscle measurements over time, and a compound symmetry (co)variance structure was assumed between residuals of the same subject (horse) in the model. Comparisons of measurements of selected paired muscles from the left and right sides were performed using paired *t*‐tests, with Bonferroni corrections. Repeated measures models, adjusting for the effect of timepoint, were constructed to investigate the effects of previous airway surgery and IMT on the change in ultrasonographic size measurements obtained. Residuals were assessed graphically for normality. The assumption of homogeneity of variance was tested by plotting the predicted values against the residual values.

For assessment of athletic performance, repeated measures models were constructed to assess relationships between explanatory variables with OR, and a compound symmetry (co)variance structure was assumed between residuals of the same subject (horse) in the model. Explanatory variables significant at the 20% level were carried forward into a backwards stepwise multivariable model. Residuals were assessed graphically for normality. The assumption of homogeneity of variance was tested by plotting the predicted values against the residual values. Significance was set at *p* < 0.05.

## 3 RESULTS

### 3.1 Population

**FIGURE 1:**
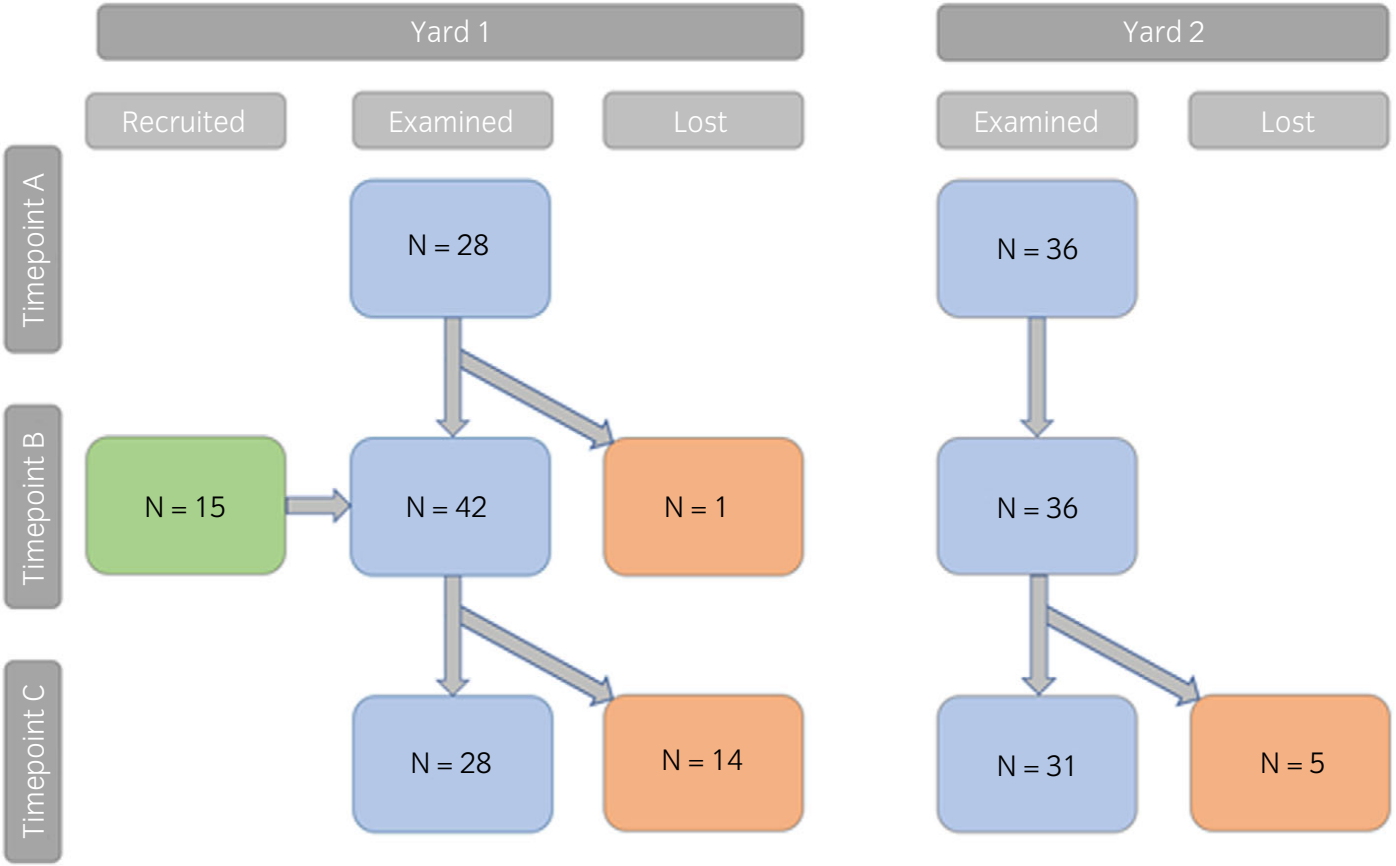
Flow chart displaying the horses that underwent ultrasound examination at yards 1 and 2 during timepoints A–C. the horses examined at each timepoint are displayed in blue; additional horses that were recruited are displayed in green; horses that were lost from the study are displayed in orange

A total of 79 horses were recruited, 43 from yard 1 (one mare and 42 geldings) and 36 from yard 2 (five mares and 31 geldings). The mean age at recruitment was 5.8 [±1.7] at yard 1 and 5.5 [±1.1] years at yard 2. The mean number of seasons in training was 3.9 [±1.8] at yard 1 and 3.1 [±1.3] at yard 2. Sixty‐four horses were examined at timepoint A (28 at yard 1, 36 at yard 2). One horse was sold from yard 1 before timepoint B but an additional 15 horses were recruited from yard 1 such that a total of 78 horses were examined at timepoint B (42 at yard 1, 36 at yard 2). Nineteen horses were removed from training between timepoint B and C, such that 59 horses were examined at timepoint C (28 at yard 1, 31 at yard 2). All horses, except the one horse sold from yard 1 before timepoint B, were examined on a minimum of two consecutive occasions. A flow chart displaying case recruitment is shown in Figure 1.

### 3.2 Horse information

Overall, 51/79 (65%) horses had undergone airway surgery and 28/79 (35%) horses had not previously undergone airway surgery (to the trainers' knowledge). In terms of yard distribution, 33/43 (77%) horses from yard 1 and 18/36 (50%) horses from yard 2 had undergone airway surgery. In terms of airway surgery type, 30/51 (59%) horses had surgery to treat soft palate dysfunction (23 = soft palate thermocautery, 7 = laryngeal tieforward and soft palate thermocautery), no horses had surgery to treat vocal fold dysfunction (ventriculocordectomy) as a standalone treatment, but 21/51 (41%) horses had combination surgery (ventriculocordectomy and soft palate thermocautery).

### 3.3 Ultrasonographic examination

Ultrasonographic examination was successfully performed in all but one horse. During examination at timepoint A, the author was unable to scan the right side of this horse as the horse was unsettled by the ultrasound machine on that side and would not stand to allow ultrasonographic images to be obtained. During subsequent examination at timepoints B and C, all ultrasonographic images, on both the left and right sides, were successfully obtained.

### 3.4 Exercise training

Information was obtained from the telephone follow‐up; the perception was that it takes approximately 12 weeks for the horses to reach race fitness following a break (inclusion criteria required a minimum of 8 weeks without exercise training). On re‐introduction to training, exercise at walk and trot was performed for 10 days to 3 weeks on average, prior to starting canter exercise. The duration and intensity of canter exercise was gradually increased over the remaining 9‐ to 10‐week period. Canter exercise was undertaken on a combination of a deep sand circular gallop and hill gallop at both yards, with fast work conducted 2 or 3 days per week. Individual training programmes were tailored to each horse, and were dependent on the horse's temperament, experience, locomotor and respiratory ‘health’, fitness level and competition schedule. Jump training was dependent on the horse's experience and both yards used regular (5–7 days/week) horse walker exercise to compliment ridden exercise. Overall, each horse undertook a consistent training programme throughout the racing season (from timepoint B to C), with no change in the run up to competition, but were given a few easier days after a race. The horses were deemed to be at ‘peak’ race fitness just before (yard 1), or just after (yard 2), the first race of the season (October).

### 3.5 Ultrasonographic muscle size—Timepoint

**FIGURE 2:**
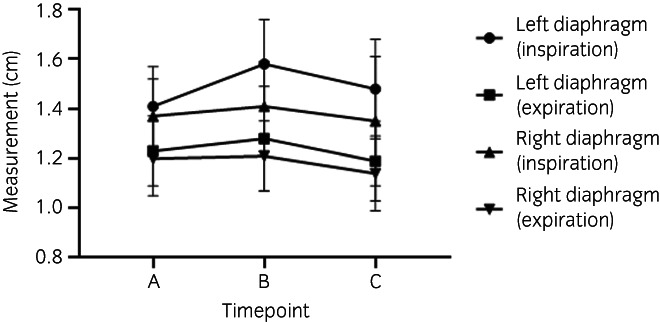
Muscle size measurements increased significantly between timepoints A and B and decreased significantly between timepoints B and C. Overall, between timepoint A and C, there was a significant increase in the diaphragm measurements on the left side during inspiration, but no significant change on the left side during expiration, or the right side during inspiration or expiration

The size measurements obtained at timepoints A, B, and C are presented in Table 1. The full statistical analysis, assessing the change in size between the different timepoints, is presented in Table S2, and represented graphically in Figure 2 and Graphs S1.

**TABLE 1:** Size measurements for each structure, obtained from both yard 1 and 2, at timepoint A‐C. Results presented as mean [± standard deviation] or median (± interquartile range)

Muscle	Side	Size (cm) Timepoint A	Size (cm) Timepoint B	Size (cm) Timepoint C
Thyrohyoideus	Left	0.32 [±0.05]	0.34 [±0.06]	0.36 [±0.06]
	Right	0.33 [±0.06]	0.32 (±0.09)	0.36 [±0.06]
Cricothyroideus (cm^2^)	Left	1.22 (±0.32)	1.22 [±0.15]	1.30 [±0.18]
	Right	1.26 [±0.16]	1.25 [±0.16]	1.31 [±0.15]
Basihyoid Bone Depth	—	1.88 (±0.44)	1.79 (±0.34)	1.80 (±0.34)
Geniohyoideus	Left	2.10 [±0.24]	2.21 [±0.24]	2.07 (±0.32)
	Right	2.10 [±0.31]	2.16 [±0.24]	2.06 (±0.32)
Genioglossus	Left	8.83 [±0.49]	8.80 [±0.47]	8.63 [±0.51]
	Right	8.98 [±0.49]	8.92 [±0.49]	8.80 [±0.56]
Sternothyrohyoideus (cm^2^)	—	3.43 (±0.84)	3.55 (±0.89)	3.57 (±0.77)
Extensor Carpi Radialis	Left	6.80 [±0.42]	6.85 [±0.49]	7.02 [±0.46]
	Right	7.10 [±0.44]	7.10 [±0.48]	7.16 [±0.45]
Gluteus Medius	Left	9.33 [±0.72]	9.61 [±0.76]	9.52 [±0.90]
	Right	8.89 [±0.75]	9.13 [±0.64]	9.40 [±0.80]
Vastus Lateralis	Left	8.33 [±0.56]	8.34 [±0.52]	8.31 [±0.52]
	Right	8.37 [±0.61]	8.45 [±0.61]	8.27 [±0.65]
Diaphragm	Left—Insp	1.41 [±0.16]	1.58 [±0.18]	1.48 [±0.20]
	Left—Exp	1.23 [±0.14]	1.28 (±0.21)	1.19 [±0.16]
	Right—Insp	1.37 [±0.15]	1.41 [±0.18]	1.35 (±0.26)
	Right—Exp	1.20 (±0.15)	1.21 [±0.14]	1.14 [±0.15]

#### 3.5.1 Upper airway muscles

Significant changes in the size of the TH (*L&R: p* < *0.001*) and CT (*L: p* = *0.001*, *R: p* = *0.02*) muscles were detected. A significant increase in size occurred with ongoing training at ‘race fitness’ from timepoint B to C (TH: *L: 5.9% p* = *0.003*, *R:12.5% p* < *0.001*; CT: *L: 6.6% p* = *0.001*, *R: 4.8% p* = *0.01*), with an increase from timepoint A to C (TH: *L: 12.5% p* < *0.001*, *R: 9.1% p* < *0.001*; CT: *L: 6.6% p* = *0.001*, *R: 4.0% p* = *0.01*). There was no significant change in the depth of the base of the lingual process of the basihyoid bone measured at any timepoint (*p* = *0.149*).

#### 3.5.2 Diaphragm

A significant change in diaphragm size was detected, measured on both the left (*Insp & Exp p* < *0.001*) and right (*Insp p* = *0.007*; *Exp p* = *0.002*). The diaphragm thickness increased during the initial 12‐week period of fitness training from timepoint A and B, during inspiration (*L: 12.1% p* < *0.001*, *R: 2.9% p* = *0.01*) and expiration (*L: 4.1% p* < *0.001*). Subsequently there was a decrease in size with ongoing training at ‘race fitness’ between timepoint B and C, during inspiration (*L: −6.8% p* = *0.001*, *R: −4.4% p* = *0.005*) and expiration (*L: −7.6% p* < *0.001*, *R: −6.1% p* = *0.001*). The diaphragm size increased between timepoints A and C on the left side during inspiration (*5.0% p* = *0.004*) and decreased during expiration on the right side (*−5.3% p* = *0.01*).

#### 3.5.3 Accessory respiratory muscles

Significant changes in the size of the GH muscles were detected (L: *p* = *0.001*; R: *p* = *0.02*). There was an increase in size from timepoint A to B (L: 5.2% *p* < *0.001*; R: 2.9% *p* = *0.03*), but there was no significant change in size between timepoint A and C (L: *p* = *0.09*; R: *p > 0.9*). No change in the left GG was observed at any time point (*p* = *0.05*) but a significant change was detected for the right GG (*p* = *0.02*). There was a significant reduction in size of the right GG from timepoints A to C (−2.0% *p* = *0.005*), with no significant change between timepoints A to B (*p* = *0.333*) and a significant reduction from timepoint B to C (−1.4% *p* = *0.04*). Overall, a significant change in the size of the STH muscle was detected (*p* < *0.001*). There was a significant increase in the size of the STH from timepoint A to C (*4.9% p* < *0.001*) with the significant increase detected in the initial 12‐week period of fitness training from timepoint A to B (*3.5% p* = *0.003*).

#### 3.5.4 Locomotor muscles

No change in the size of the right ECR was detected at any timepoint (*p* = *0.1*), whereas a change in the size of the left ECR was detected (*p* < *0.001*). There was a significant increase in the size of the left ECR from timepoint B to C (2.5% *p* < *0.001*) with an increase from timepoint A to C (3.2% *p* < *0.001*). Comparisons of the measurements obtained from the left and right sides showed that, despite the increase in the size of the left ECR, the right ECR was significantly larger at both timepoint A (*p* < *0.001*) and at timepoint C (*p* < *0.001*).

Overall, significant changes in the size of the left and right GM were detected (*p* < *0.001*). There was a significant increase in the size of the GM from timepoint A to C (*L: 2.0% p* = *0.003*, *R: 5.7% p* < *0.001*), with the significant increase in the initial 12‐week period of fitness training from timepoint A to B (*L: 3.0% p* < *0.001*, *R: 2.7% p* < *0.001*). There was a further significant increase with ongoing training at ‘race fitness’ from timepoint B to C on the right (*3.0% p* = *0.002*). Comparisons of the measurements obtained from the left and right sides showed that the left GM was significantly larger than the right GM at timepoint A (*p* < *0.001*), but that there was no significant difference in the size of the left and right GM at timepoint C (*p* = *0.1*).

No change in the size of the left VL was detected at any timepoint (*p* = *0.7*). A change in the size of the right VL was detected (*p* = *0.04*), with a significant decrease in size from timepoint B to C (−2.2% *p* = *0.01*). However, there was no significant difference between timepoint A and C (*p* = *0.2*).

Five patterns of change in measurements over time emerged; these are illustrated in Figure 2 and Graphs S1.

### 3.6 Ultrasonographic muscle size—Inspiratory muscle training

Results of the repeated measures model analyses, adjusted for the effect of timepoint, are summarised in Table S3. Inspiratory muscle training was performed at yard 1 only.

#### 3.6.1 Upper airway muscles

There was no significant association between IMT and the measurement of the TH (L: *p* = *0.23*, R: *p* = *0.3*) and CT (L: *p* = *0.2*, R: *p* = *0.8*) muscles, or the depth of the lingual process of the basihyoid bone (*p* = *0.7*).

#### 3.6.2 Diaphragm

There was a significant interaction between IMT and diaphragm size on the left and right side, during inspiration and expiration (*Left: Insp p* = *0.01*; *Exp p* = *0.01*; *Right: Insp p* = *0.003*; *Exp p* = *0.007*). For the diaphragm on the left side during inspiration there was a significant increase in size in the high‐load (treatment) group (*1.3% p* < *0.001*), whereas there was no significant change in size in the low‐load (control) group (*p* = *0.8*). There was no significant change in the other measurements for the high‐load treatment group (*Left: Exp p* = *0.1*; *Right*: *Insp: p* = *0.3*, *Exp p* = *0.7*), but a significant decrease in size in the low‐load (control) group (*Insp: R: −4.9% p* = *0.002*, *Exp: L: −9.3% p* = *0.04*, *R: −5.0% p* < *0.001*). In summary, high‐load IMT either maintained or significantly increased the size of the diaphragm, whereas there was no change or a significant reduction in size in the low‐load group.

#### 3.6.3 Accessory respiratory muscles

There was a significant interaction between IMT and STH size (*p* = *0.02*). There was a significant increase in size in the high‐load (treatment) group (*5.9% p* = *0.001*), whereas there was no significant change in size in the low‐load (control) group (*p > 0.9*). There was no significant association between IMT and the measurement of the GH (L: *p > 0.9*, R: *p* = *0.1*) and GG (L: *p* = *0.2*, R: *p* = *0.2*) muscles.

#### 3.6.4 Locomotor muscles

There was no significant association between IMT and the measurement of the ECR (L: *p* = *0.4*, R: *p* = *0.8*), GM (L: *p* = *0.2*, R: *p* = *0.536*) and VL (L: *p* = *0.9*, R: *p* = *0.7*) muscles.

### 3.7 Ultrasonographic muscle size—Airway surgery

Results of the repeated measures model analyses, adjusted for the effect of timepoint, are summarised in Table S5. There was no significant effect of airway surgery on the measurements of any of the respiratory muscles (upper airway, diaphragm, or accessory respiratory muscles).

#### 3.7.1 Locomotor muscles

There was a significant interaction between airway surgery status and the size of the left and right GM (*p* = *0.03* and *p* = *0.04* respectively). For the left GM, there was a significant increase in the size in horses that had not undergone airway surgery (*3.9% p* < *0.001*), whereas there was no change in size for the horses that had previously undergone airway surgery (*p* = *0.3*). For the right GM, there was a significant increase in size in both the airway surgery (4.5%) and non‐airway surgery (*7.4% p* < *0.001*) groups, but the increase was significantly greater for the group that had not undergone airway surgery (*p* < *0.04*). In summary, the increase in GM size was significantly greater in horses that had not undergone airway surgery.

Further assessment of the interaction between airway surgery and yard was explored, there was no significant association between airway surgery and yard for the size measurements of the left and right GM (*p* = *0.8* and *p* = *0.8*, respectively), indicating that the influence of airway surgery on measurements cannot be explained by training differences between the yards, or vice versa.

### 3.8 Athletic performance

Overall, 69 horses were examined at timepoint B (Yard 1 = 33, Yard 2 = 36) and 81 horses at timepoint C (Yard 1 = 50, Yard 2 = 31). An OR is assigned for every race by the British Horseracing Authority (BHA) official handicappers once the horse has taken part in three races under rules, as such, an OR was not available to be assigned for every horse at each examination. An OR was assigned to 45/69 horses assessed at timepoint B, and 57/81 horses measured at timepoint C. The results of the univariate repeated measures linear model analysis are presented in Table 2. Parameters with a *p* value < 0.2 were carried forward to the multivariable model and included: yard, IMT, number of seasons in training, weight, and left diaphragm thickness during inspiration. The results of the final multivariable model are presented in Table 3. There was a significant association between yard (Yard 1: mean [±SD] OR = 121 [±18]; Yard 2 = 140 [±14]; *p* < *0.001*), and number of seasons in training (*p* < *0.001*) and the OR at the time of examination. No significant independent association between diaphragm thickness on the left side during inspiration and OR was detected (*p* = *0.07*).

**TABLE 2:** Results from the repeated measures linear model comparing the official rating

Variable	Estimate	95% CI for estimate	SE	*p*
Yard	−19.09	−27.02 to −11.16	3.97	<*0.001* *
Airway surgery status	3.82	−6.57 to 14.20	5.17	*0.5*
IMT	−4.70	−10.94 to 1.54	3.12	*0.2* *
Number of seasons in training	3.80	1.12 to 6.47	1.34	*0.006* *
Weight	0.20	0.08 to 0.32	0.06	*0.002* *
Diaphragm—Left Inspiration	14.51	1.34 to 27.68	6.58	*0.03* *
Gluteal Muscle—Left	−1.35	−5.04 to 2.33	1.85	*0.5*
Rump Fat Thickness	12.15	−115.39 to 139.68	62.69	*0.9*
IMSi	0.28	−0.48 to 1.04	0.36	*0.5*

Abbreviations: CI, confidence interval; IMSi, Index of Inspiratory Muscle Strength; IMT, Inspiratory Muscle Training; SE, standard error.

* Significance at *p* < 0.2.

**TABLE 3:** Results from the multivariable model

Variable	Estimate	95% CI for estimate	SE	*p*
Yard	−27.42	−36.71 to −18.14	4.63	<*0.001* *
Number of seasons in training	5.52	3.04 to 8.00	1.23	<*0.001* *
*Diaphragm*—*Left Inspiration*	*16.11*	*−1.36 to 33.57*	*8.74*	*0.07*

Abbreviations: CI, confidence interval; SE, standard error.

* Significance at *p* < 0.05.

## 4 DISCUSSION

The equine respiratory and locomotor muscles respond to conventional exercise training by increasing in size. The significant changes in diaphragm size associated with high‐load IMT are interesting and noteworthy, reflecting the findings from studies conducted in human athletes. The association between airway surgery status and gluteal muscle size was unexpected and warrants further investigation.

The focus of this investigation was the response of the diaphragm to training. The results presented here are the first to show that the thickness of the equine diaphragm increases following 12 weeks of conventional exercise training. This finding reflects the results of investigations conducted in human athletes, showing that an increase in diaphragm thickness was observed following strength training and conditioning.^7^ Subsequently, the results presented here demonstrated a reduction in equine diaphragm thickness with ongoing training at race fitness; the reason for this is unknown. One aspect could be a relative training deficit, with reduced stimulus on the diaphragm during maintenance exercise training, leading to a detraining response. Another explanation could be that the initial increase in diaphragm size is subsequently remodelled, whereby the functional benefits are maintained despite a decrease in thickness with ongoing race training. Indeed, the results presented in a linked investigation (L. Fitzharris et al., unpublished data) showed that inspiratory muscle strength was unchanged between timepoint B and C in the low‐load group, despite the reduction in diaphragm size. Overall, the changes in diaphragm thickness observed in the racehorse may suggest that the initial 12‐weeks of fitness training stimulates a strength training response with an increased thickness, potentially associated with an increase in type II muscle fibres with a large cross‐sectional area as has been shown with the gluteus medius,^21^, ^22^ whereas ongoing training at race fitness may stimulate an endurance training response with an increase in type I muscle fibres^22^, ^23^ with a small cross‐sectional area leading to a reduction in thickness but maintained function.

High‐load IMT was associated with an increased, or maintained, diaphragm thickness, compared with the reduction in diaphragm thickness observed in the low‐load IMT group and no IMT group (yard 2). The increase in diaphragm thickness with high‐load IMT is consistent with the results from investigations conducted in human subjects^8^ and in rat models, the latter of which demonstrated hypertrophy of both type I and type IIa diaphragm muscle fibres in response to IMT.^24^, ^25^ In addition to an increase in diaphragm size reported in this study, the results presented in a linked investigation (L. Fitzharris et al., unpublished data) showed an increase in inspiratory muscle strength in response to both conventional race training (timepoint A to B) and high‐load IMT (timepoint B to C). This indicates that a change in diaphragm function is occurring in association with the change in structure.

The diaphragm thickness measurements obtained on the left side were larger than the right side, during both inspiration and expiration, at all timepoints. The reason for this difference is unknown. The abdominal viscera adjacent to the diaphragm on the left and right sides is different. The liver situated on the right side is relatively ‘immobile’ whereas the stomach on the left side is more mobile and could apply different pressure leading to differential contraction and therefore thickness of the diaphragm in situ.^20^ An alternative explanation is thoracic asymmetry during locomotion at canter and gallop with paradoxical motion of the left and right sides^26^ leading to a different pressure applied to, and subsequent contraction strength of, each side of the diaphragm and a resultant difference in thickness. However, measurement of the diaphragm post‐mortem did not show a significant difference between measurements obtained on the left and right side.^27^

In contrast to the response of the diaphragm, the upper airway muscle (CT and TH) size increased in response to ongoing training at race fitness between timepoint B and C. This finding indicates that the exercise stimulus during ongoing training at race fitness is sufficient to induce an increase in muscle size. The CT and TH muscles both play a role in stabilisation of the larynx during exercise; the increase in size from timepoint B to C may indicate that strenuous exercise, associated with galloping and jumping, induces hypertrophy of these muscles. The CT muscle is associated with vocal fold tension and dysfunction is thought to play a role in some cases with vocal fold collapse.^28^ The TH is associated with laryngohyoid position, with rostral advancement of the larynx, and is thought to play a role in intermittent dorsal displacement of the soft palate (iDDSP).^29^ Interestingly, a common management tool for iDDSP is to increase fitness,^30^, ^31^, ^32^ the results here indicate an increase in TH size—further research is required to determine whether this change in size is associated with an improvement in iDDSP. The use of fitness training in cases with vocal fold collapse is more difficult to interpret due to the relationship with left arytenoid cartilage collapse, associated with recurrent laryngeal neuropathy, which is a degenerative condition that will not respond to an exercise stimulus. The results presented here did not show an association between upper airway muscle measurements and airway surgery status; however, it should be noted that the upper airway function of these horses is unknown as categorisation was based on airway surgery status rather than direct assessment (e.g. exercising endoscopy was not performed). Further investigation into the association between CT and TH muscle size and upper airway function is warranted.

The interaction between the change in size of the diaphragm and muscles of the upper airway is interesting, with the size of the diaphragm increasing in the initial training period (timepoint A to B), while the upper airway muscles increase in size later with ongoing training at race fitness (timepoint B to C). This indicates that the increase in diaphragm size occurs in advance of the increase in the size of the upper airway muscles.

There was no significant change in the size measurement of the upper airway muscles in response to IMT; however, as discussed above, there was an increase in size in response to whole‐body training. This raises the question as to whether whole‐body training may be better than IMT for training the upper airway. In a previous investigation assessing the change in upper airway function in response to IMT, using dynamic endoscopy, there was a reduction in the severity of vocal fold collapse, palatal instability and intermittent dorsal displacement of the soft palate.^13^ Similar results were reported in a likewise investigation conducted in human athletes assessing the effect of IMT on exercise‐induced laryngeal obstruction,^33^ which indicates that IMT may have a beneficial training effect on the muscles of the upper airway resulting in improved function.^13^, ^33^ However, a recent investigation conducted in human subjects compared the improvement in upper airway function in response to the provision of information on breathing advice (IBA) alone or in conjunction with IMT.^34^ The results indicated that IBA alone had an equivalent benefit on long‐term upper airway function when IBA was combined with IMT.^34^ Unfortunately, the application of IBA is not translatable to the horse. It remains unclear whether IMT has a role in the treatment of upper airway disorders and if so, what the optimal IMT programme may be.

The response of the accessory respiratory muscles to training is harder to interpret. The GG and GH muscle measurement results are slightly compounded by the fact that the measurement of these two adjacent muscles is closely associated. In addition, both the GG and GH are extrinsic muscles of the tongue and play a role in mastication, with secondary roles in respiration. Therefore, changes in the dietary intake may influence the size of these muscles. Investigations in human athletes have shown that both the GH and GG are activated during IMT^35^ however, further research is required to fully assess the response of these muscles to both conventional exercise training and IMT in the horse.

Thickness measurement of the ECR and GM locomotor muscles at timepoint A, following an 8‐week period of rest, was asymmetrical. Subsequent exercising training reduced the degree of asymmetry in the ECR, and the GM thickness became symmetrical. The right ECR was thicker than the left at all timepoints, despite the left ECR increasing with training. In contrast, the left GM was thicker initially, but there was no difference in GM thickness between the left and right sides at timepoint C. These results indicate that conventional exercise training promotes more symmetrical locomotor muscling. Further research investigating the response to training is required, such as determining the potential effect of canter and gallop footfall on skeletal muscle thickness of the associated limbs.

The interaction between airway surgery status and gluteal muscle thickness was interesting and unexpected. The results indicate that horses that had not previously undergone airway surgery had significantly greater gluteal muscle measurements. This finding suggests that previous, or current, upper airway compromise may have an influence on locomotor muscle conditioning. Only information on airway surgery was collected; examination of upper airway function was not undertaken in this investigation. Further research is required to identify whether there is a difference in how the two groups of horses are trained (e.g. horses with upper airway compromise undertake more exercise on the flat compared with the hill gallop), or whether there is a difference in how the horses respond to training (e.g. a horse with upper airway dysfunction shows a reduced response to exercise training).

The results of the preliminary univariate analysis of race performance suggest that there may be an association between the measurement of diaphragm thickness on the left side during inspiration and the OR at the time of examination, indicating that diaphragm thickness may contribute to determinants of performance. However, this association between diaphragm thickness and race performance did not remain significant in the multivariable analysis. This study was not designed for the analysis of race performance and as such is likely to be underpowered. However, these results are intriguing and warrant further investigation in a larger population, with additional comparisons between Thoroughbred racehorses trained for flat and National Hunt racing.

The assessment of race performance is challenging, with multiple parameters and factors to take into consideration. The investigation by Young et al^18^ showed a significant association between left ventricular dimensions and race performance and was conducted in a similar population of horses. However, that study assessed 482 horses and therefore was adequately powered to perform robust analysis, and slightly different performance parameters were assessed.^18^ The OR was chosen as the parameter to denote athletic performance in this investigation because it provides an overview of the quality of horse and reflects the horse's rating going into the race, providing a trend for performance. Measurement of diaphragm thickness in a larger number of horses, along with measurements of left ventricular size, is warranted to assess the association between cardiac, respiratory, and locomotor muscle function and athletic performance. In addition, examination of horses in training for competition in flat races would be of interest, as there may be a stronger association between muscle size and sprint performance.

The main limitations of this investigation are the small number of horses included in the final data set that underwent IMT and that all ultrasonographic measurements were performed by a single observer. Strict protocols were followed to obtain repeatable measurements, with previous investigations confirming that the ultrasonographic thickness measurement protocols used were repeatable.^19^, ^20^ However, operator error could have led to differences in the muscle size measurements, both between the left and right sides within a timepoint, or of the same muscle between the different timepoints. A low number of horses were assessed in the preliminary sub‐analysis of race performance, as such the investigation is underpowered and firm conclusions cannot be drawn. Finally, the level of detraining prior to entry into the study may have been incomplete. A period of 8 weeks was selected as the minimum inclusion criteria to allow the physiological changes from the previous season's conditioning to reverse while maximising case recruitment. This could explain why there was no change in the size measurement of some muscles between timepoints A and B.

## 5 CONCLUSION

Overall, ultrasonographic examination is an easy and non‐invasive method to obtain information about skeletal muscle changes in response to a training stimulus. Conventional exercising training increases the thickness of both locomotor and respiratory muscles in the Thoroughbred racehorse. In addition, IMT increases the thickness of the diaphragm. The association between diaphragm thickness and race performance warrants further investigation in a larger number of horses, along with locomotor muscle and cardiac measurements.

## AUTHOR CONTRIBUTIONS

L.E. Fitzharris executed the study. All authors were involved in the study design, data analysis, preparation and final approval of the manuscript.

## FUNDING INFORMATION

Horserace Betting Levy Board (HBLB; Grant number CS022).

## CONFLICT OF INTEREST

A.K. McConnell has previously developed two commercial devices for inspiratory muscle training in human subjects but no longer has any financial interest in either product. Other authors declare no competing interests.

### PEER REVIEW

The peer review history for this article is available at https://publons.com/publon/10.1111/evj.13598.

## ETHICAL ANIMAL RESEARCH

University of Bristol animal welfare and ethical approval board approval (VIN/13/027) was obtained for the use of the horses and procedures in this study.

## INFORMED CONSENT

Trainers gave consent for their animals' inclusion in the study.

## Supporting information


**Graphs S1** Muscle size measurements.

**Appendix S1: Methods S1.** Ultrasound protocols

**Table S1** Inspiratory muscle training schedule.

**Table S2** Results from the repeated measures model comparing the muscle size measurements at each timepoint, for the measurements obtained from yard 1 and yard 2.

**Table S3** Results from the univariate model, adjusted for timepoint, to assess the effect of inspiratory muscle training on the ultrasound size measurements.

**Table S4** Results from the univariate model, adjusted for timepoint, to investigate the effect of yard on the ultrasound size measurements.

**Table S5** Results from the univariate model, adjusted for timepoint, to assess the effect of airway surgery on the ultrasound size measurements.

## Data Availability

The data that support the findings of this study are available from the corresponding author upon reasonable request.
