# Supplementary material for: Training the equine respiratory muscles: Ultrasonographic measurement of muscle size
Source: Equine Vet J. 2022 Jun 19;55(2):295–305. doi: 10.1111/evj.13598 (PMC10084327; doi:10.1111/evj.13598)
Supplement: Supplementary file 1 — Graphs S1 Muscle size measurements. [file EVJ-55-295-s007.pdf]

Graphs S1:

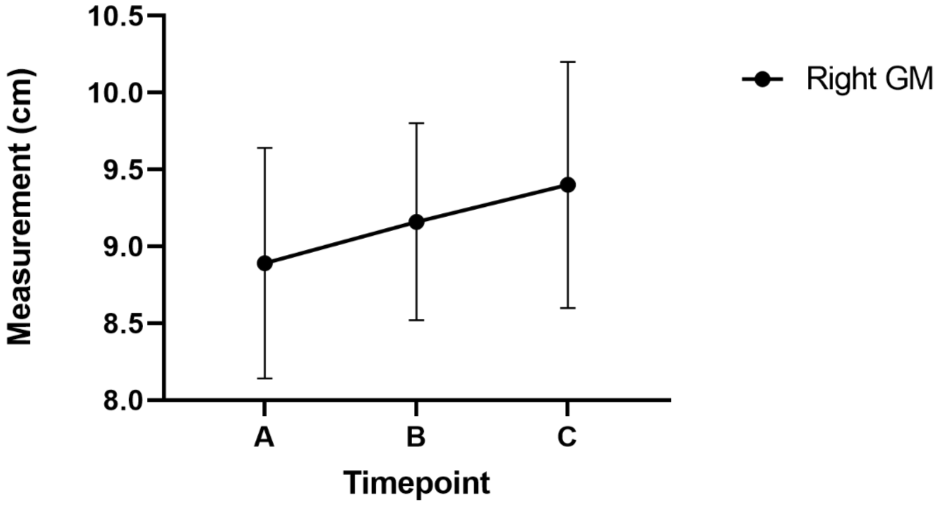

Graph 1 Muscle size measurements which increased significantly between all timepoints (A to B, B to C, and A to C).

GM: Gluteus medius

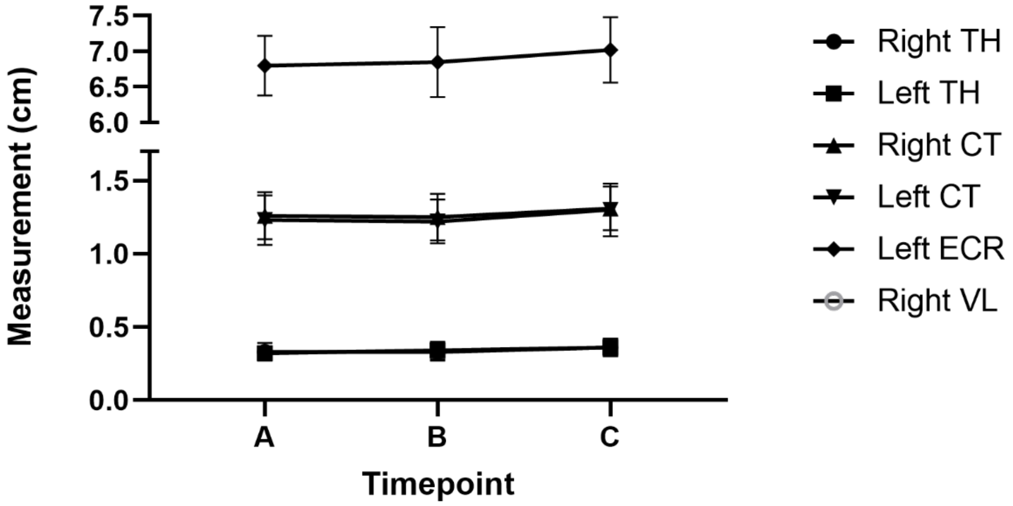

Graph 2 Muscle size measurements did not change significantly from timepoint A to B, then increased significantly in size from timepoint B to C. Overall, there was a significant increase from timepoint A to C for the left and right TH, the left and right CT and the left ECR; but no change was detected between timepoints A and C for the right VL.

TH: Thyrohyoideus; CT: Cricothyroideus; ECR: Extensor carpi radialis; VL: Vastus lateralis.

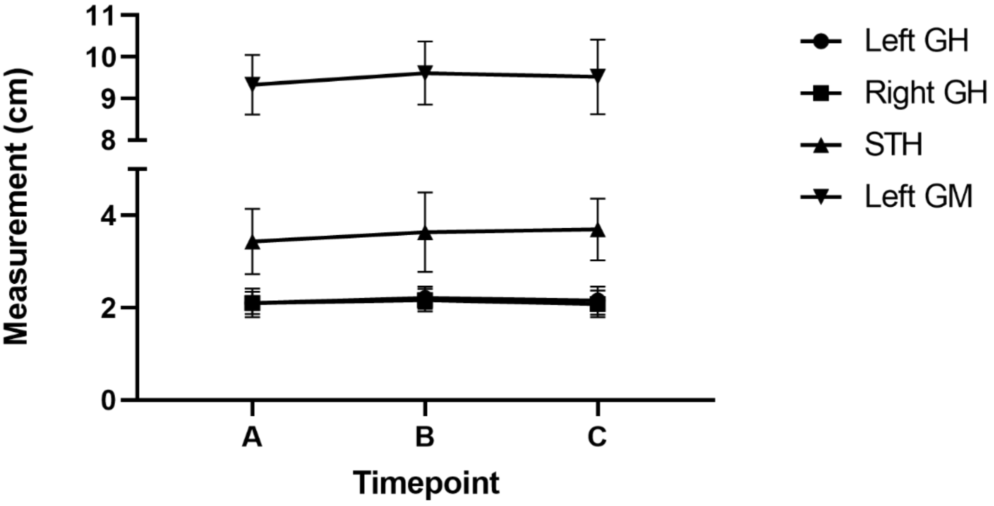

Graph 3 Muscle size measurements increased significantly between timepoints A and B with no significant change between timepoints B and C. Overall, there was a significant increase between timepoints A and C for STH and left GM; but no change was detected between timepoints A and C for left and right GH.

GH: Geniohyoideus; STH: Sternothyrohyoideus; GM: Gluteus medius.

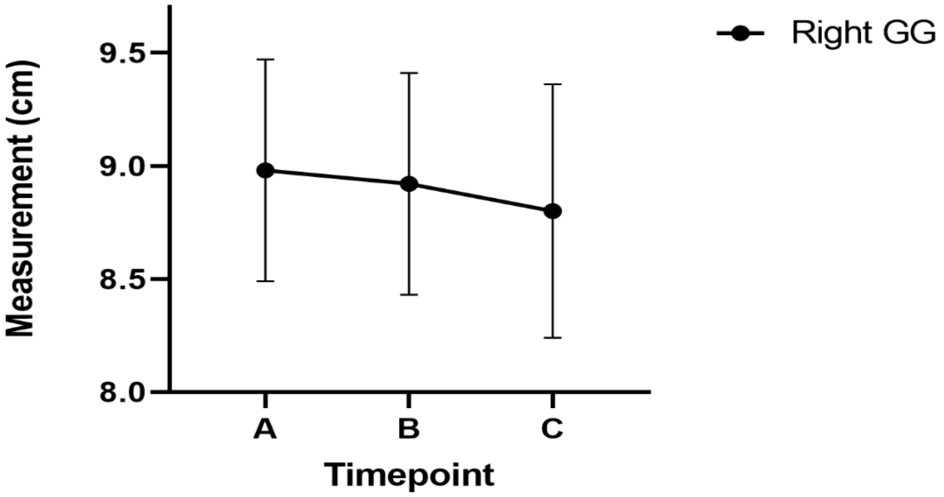

Graph 4 Muscle size measurements did not change significantly between timepoints A and B, but measurements decreased significantly between timepoints B to C. Overall, there was a significant decrease from timepoint A to C.

GG: Genioglossus.
