# Supplementary material for: Training the equine respiratory muscles: Ultrasonographic measurement of muscle size
Source: Equine Vet J. 2022 Jun 19;55(2):295–305. doi: 10.1111/evj.13598 (PMC10084327; doi:10.1111/evj.13598)
Supplement: Supplementary file 2 — Appendix S1: Methods S1. Ultrasound protocols [file EVJ-55-295-s001.pdf]

Equine Veterinary Journal BEVA

Methods S1: Ultrasound protocols

DIAPHRAGM

**Description**	Transducer placed longitudinally in the 10th intercostal space with the top of transducer positioned 1cm below the line between the cranioventral aspect of the tuber coxae and the proximal margin of the olecranon. Record two 30s prospective cine-loop (to contain a minimum of 3x inspiration and expiration).
**Image Measurement**	Each cine-loop was reviewed frame by frame, and still images of peak inspiration and peak expiration were obtained and saved for measurement. Measurements were performed on the ultrasound machine using the electronic callipers, with the diaphragm thickness measured from inside edge of the superficial border to the inside edge of the deep border, in the middle of the screen, denoted by an orange triangle automatically present on the ultrasound image. Measurement was only performed for images in which the diaphragm could be easily identified, with a clear distinction between inspiration and expiration.
**Ultrasound Machine Settings**	Transducer: Linear<br>Frequency: 13MHz<br>CHI: On<br>Gain: 46<br>Gray Map: D<br>Depth: 6cm<br>Focus Points: 2 (depth ~2cm and ~4cm)<br>Dynamic Range: 69<br>Frame rate: 25 frames/second
Example ultrasound image and measurement of the diaphragm, internal intercostal and external intercostal muscles.	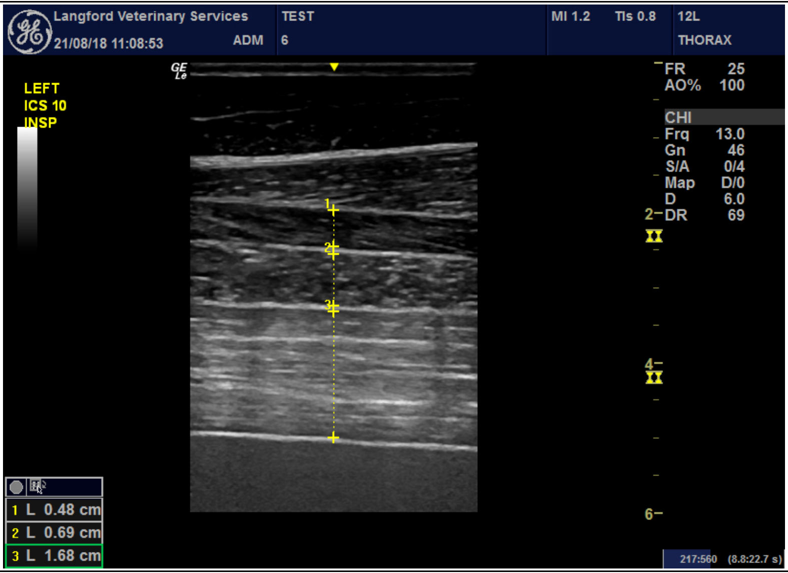

Photograph to demonstrate ultrasound transducer placement, for examination of the diaphragm, within the 10th intercostal space, 1cm below a marker placed on a line between the cranioventral aspect of the tuber coxae and the proximal margin of the olecranon, on the left side of the horse.	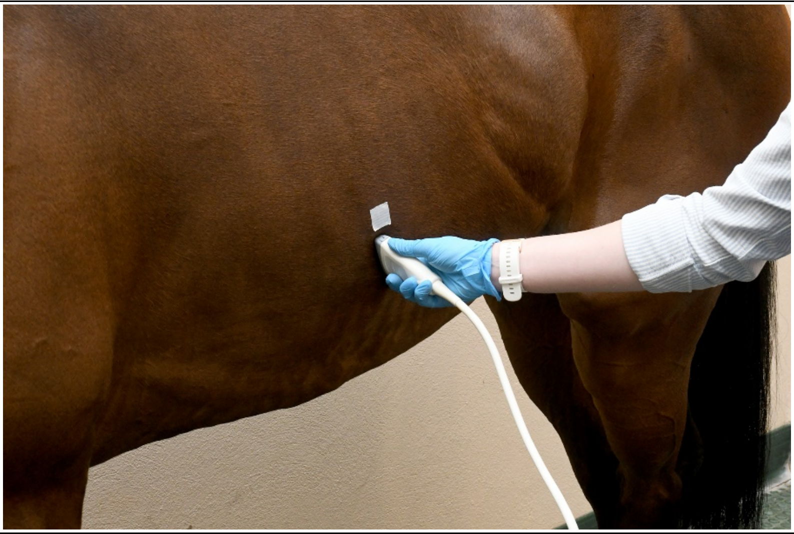

THYROHYOIDEUS

**Description**	Horse standing with head straight and slightly extended. Position the transducer horizontally at the dorsal-mid aspect of the larynx, with the rostral aspect of the transducer in contact with/underneath the caudal aspect of the mandible so you can see the thyroid, cricoid cartilage articulation with a gap in between. The caudal border of the thyroid cartilage should be in the middle of the screen. Slide the transducer ventrally if the cricoid and thyroid cartilages are touching or the thyrohyoideus muscle cannot be observer clearly.
**Image Measurement**	Measure the depth of the TH muscle from the most superficial portion of the thyroid cartilage to the adipose tissue border.
**Ultrasound Machine Settings**	Transducer: Hockey Stick<br>Frequency: 16MHz<br>CHI: off<br>Gain: 55<br>Gray Map: D<br>Depth: 3cm<br>Focus Points: 2<br>Dynamic Range: 72
Example ultrasound image and measurement of the thyrohyoideus muscle using protocol 1.	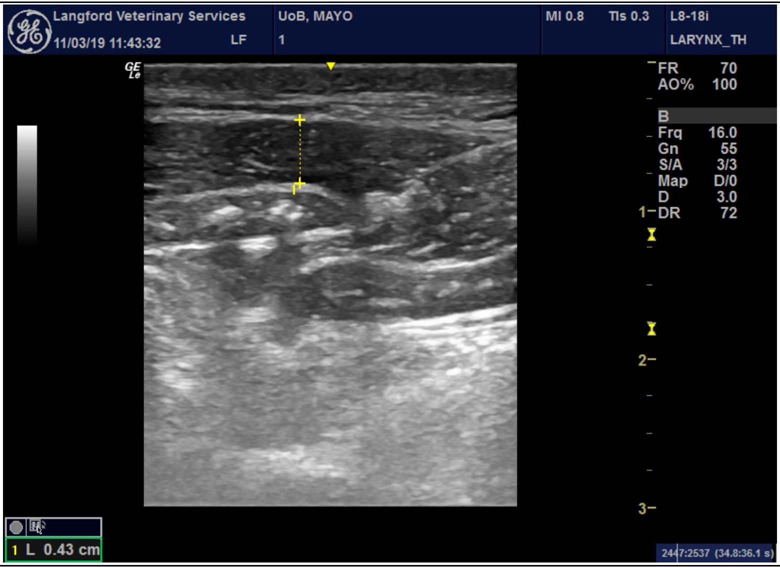
Photograph to demonstrate ultrasound transducer placement for the thyrohyoideus muscle.	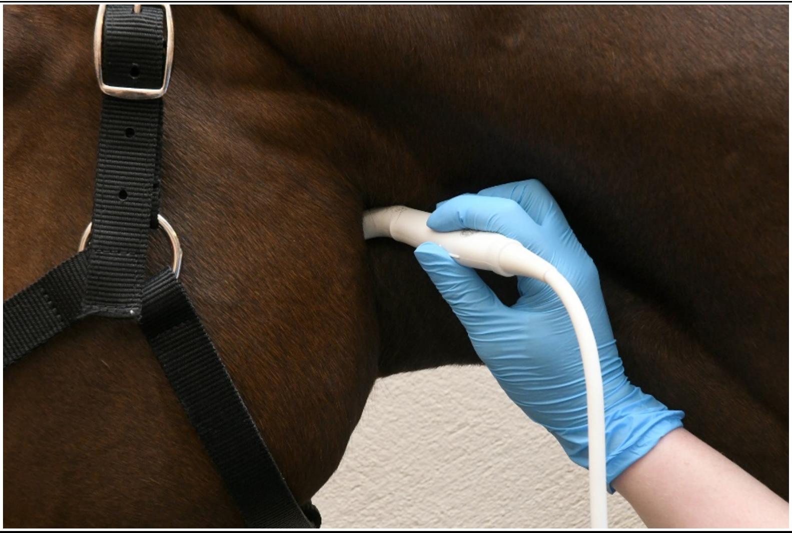

CRICOTHYROIDEUS

**Description**	Horse standing with head straight and slightly extended. Position transducer horizontally at the caudolateral window, with the rostral aspect of the transducer close/in contact with the caudal aspect of the mandible so you can see the thyroid, cricoid and arytenoid cartilages. Slide the transducer ventrally until there is a gap between the thyroid cartilage and cricoid cartilages.
**Image Measurement**	Trace the circumference of the muscle lateral to cricoid cartilage to obtain the cross sectional area.
**Ultrasound Machine Settings**	Transducer: Hockey Stick<br>Frequency: 16MHz<br>CHI: off<br>Gain: 55<br>Gray Map: D<br>Depth: 3cm<br>Focus Points: 2<br>Dynamic Range: 72
Example ultrasound image and measurement of the cricothyroideus.	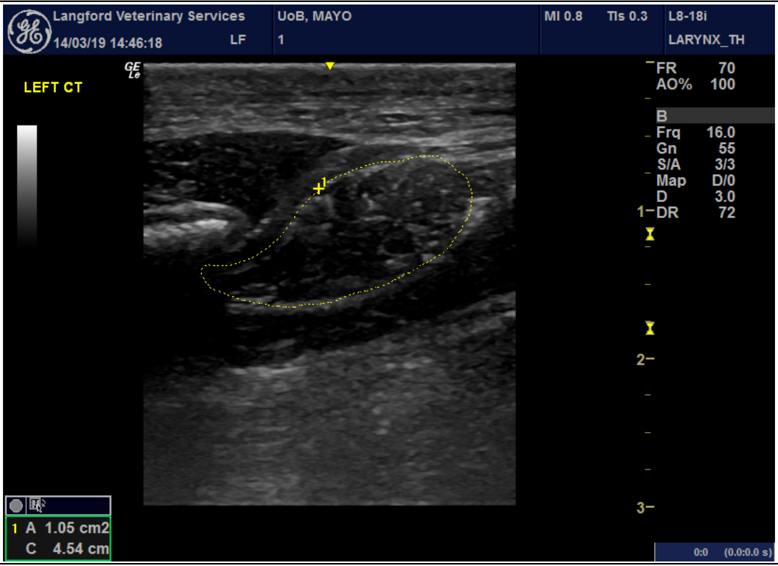
Photograph to demonstrate ultrasound transducer placement for the cricothyroideus.	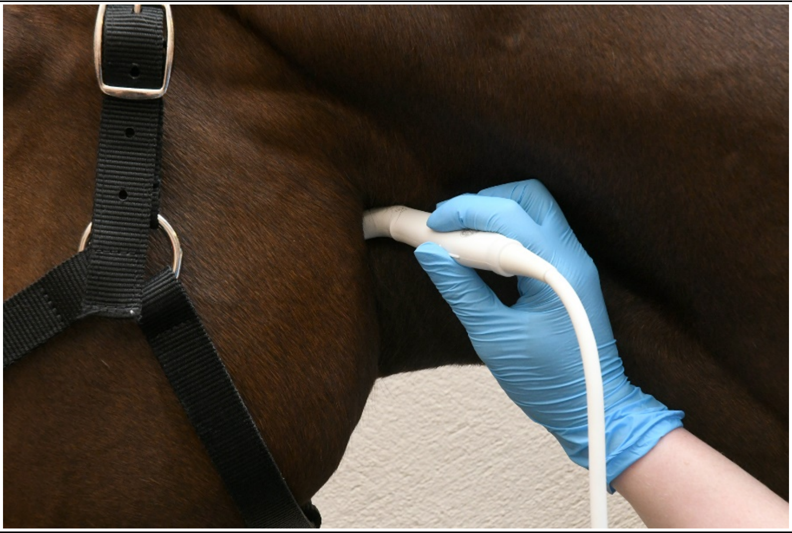

BASIHYOID BONE

<table>
<tr><td>Description</td><td>Position transducer transversely across the ventral aspect of the larynx and record cine-loop while scanning from the caudal border of the basihyoid bone to the rostral tip of the lingual process.</td></tr>
<tr><td>Image Measurement</td><td>Go through cine-loop and select images at the junction between the base of the lingual process and the basihyoid bone where ‘U’ shaped dips are observed either side of the lingual process. Measure from the adipose/connective tissue junction to the BH bone, at the intersection with the fascial plane.</td></tr>
<tr><td>Ultrasound Machine Settings</td><td>Transducer: Linear
Frequency: 13MHz
CHI: on
Gain: 54
Gray Map: D
Depth: 4cm
Focus Points: 2
Dynamic Range: 69</td></tr>
<tr><td>Example ultrasound image at the junction between the base of the lingual process and the basihyoid bone.</td><td>
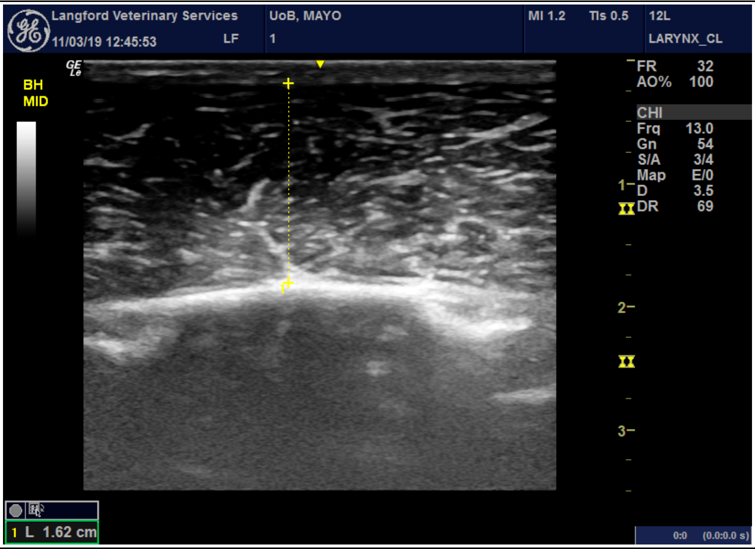

</td></tr>
<tr><td>Photograph to demonstrate ultrasound transducer placement for transverse images of the basihyoid bone.</td><td>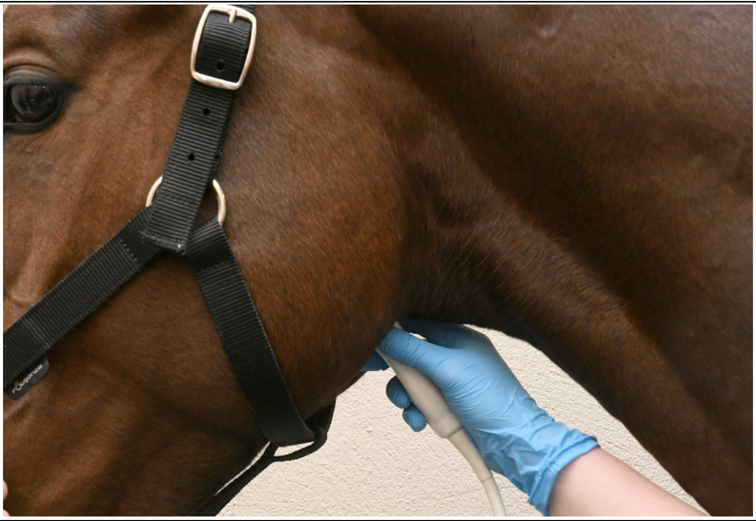</td></tr>
</table>

GENIOHYOIDEUS & GENIOGLOSSUS

**Description**	Horse relaxed and not chewing. Position transducer longitudinally between the left and right mandible with the middle of the transducer in line with the junction between the curved and straight part of the mandible. Place the transducer to the left or right of the sagittal plane to measure the left and right muscles respectively. Obtain clear image of both muscles, with blood vessel running through genioglossus at ~6cm deep.
**Image Measurement**	Geniohyoideus: Measure from the skin surface to the junction with the genioglossus.<br>Genioglossus: Measure from the junction with the geniohyoideus and the soft palate.
**Ultrasound Machine Settings**	Transducer: Linear<br>Frequency: 8MHz<br>CHI: on<br>Gain: 45<br>Gray Map: F<br>Depth: 12cm<br>Focus Points: 2<br>Dynamic Range: 69
Example ultrasound image and measurement of the geniohyoideus and genioglossus muscles.	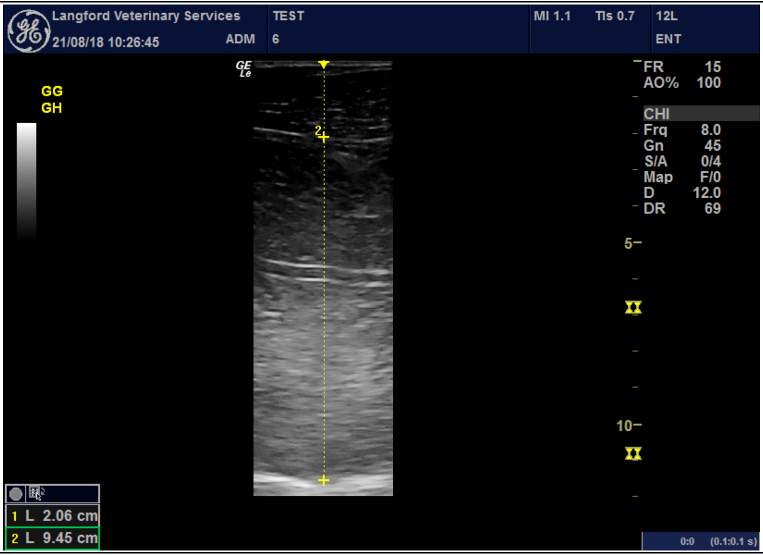
Photograph to demonstrate ultrasound transducer placement for the geniohyoideus and genioglossus muscles.	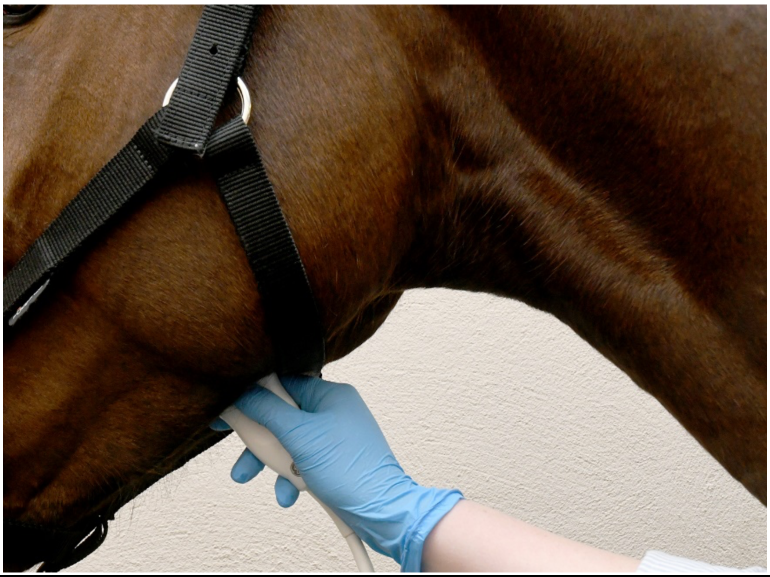

STERNOTHYROHYOIDEUS

**Description**	Horse's head in a neutral position with chin approximately at level of shoulder (relaxed as possible) and the front legs square. Position transducer at the base of the neck where it joins the body. Scan the sternothyrohyoideus muscle in transverse section between the left and right carotid arteries adjacent to the trachea.
**Image Measurement**	Trace the circumference of the sternothyrohyoideus muscle to measure the cross sectional area.
**Ultrasound Machine Settings**	Transducer: Linear<br>Frequency: 12MHz<br>CHI: on<br>Gain: 55<br>Gray Map: F<br>Depth: 8cm<br>Focus Points: 1<br>Dynamic Range: 69
Example ultrasound image and measurement of the sternothyrohyoideus.	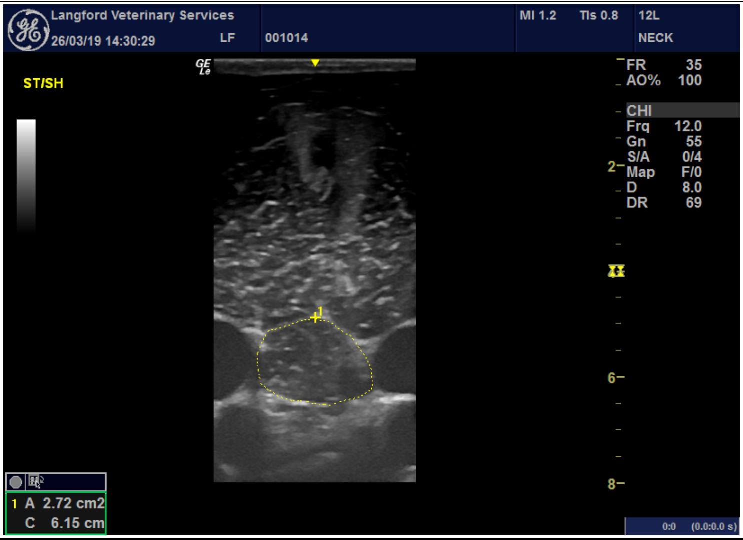
Photograph to demonstrate ultrasound transducer placement for the sternothyrohyoideus.	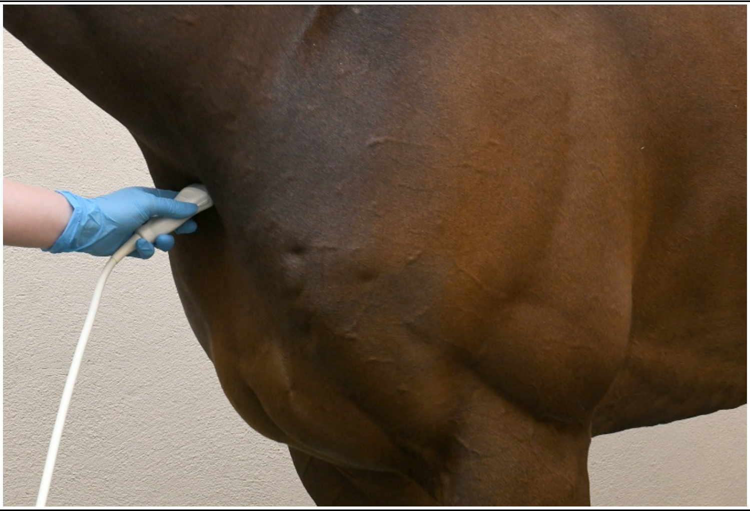

EXTENSOR CARPI RADIALIS

**Description**	With the horse standing square, position the transducer on the ECR at the point of maximal thickness with the transducer in transverse alignment with minimal pressure application. Position image such that the internal tendon crosses the middle of the screen, and the radius is horizontal.
**Image Measurement**	Measure from the middle of the screen from adipose/muscle tissue interface to radius, or to the edge of the blood vessel superficial to the bone surface if present.
**Ultrasound Machine Settings**	Transducer: Linear<br>Frequency: 12MHz<br>CHI: on<br>Gain: 53<br>Gray Map: F<br>Depth: 8cm<br>Focus Points: 1<br>Dynamic Range: 69
Example ultrasound image and measurement of the extensor carpi radialis muscle.	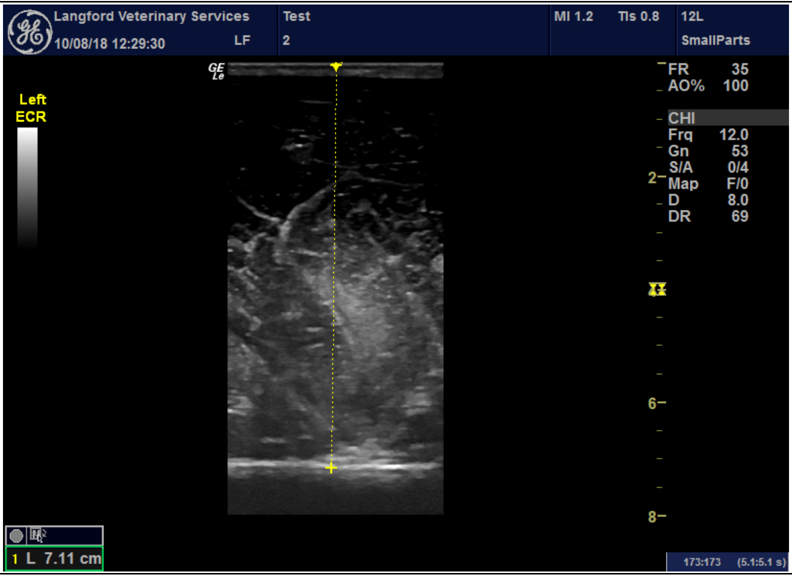
Photograph to demonstrate transducer placement for ultrasound imaging and measurement of the extensor carpi radialis muscle.	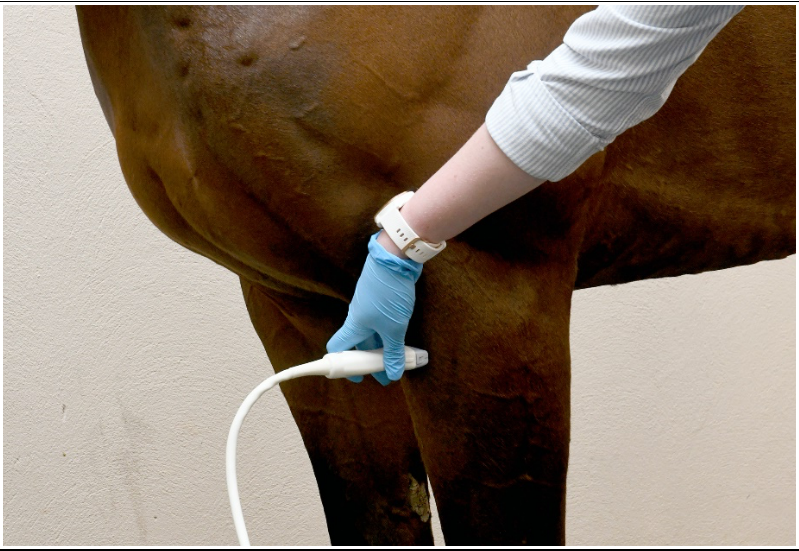

GLUTEUS MEDIUS

**Description**	Horse standing square, position a marker above the line 1/3 distance from the dorsal aspect of the tuber coxae to the tail head. Position the transducer vertically, just ventral to the marker with minimal pressure application.
**Image Measurement**	In the middle of the screen, measure from the adipose/muscle interface to the bone surface
**Ultrasound Machine Settings**	Transducer: Linear<br>Frequency: 8MHz<br>CHI: on<br>Gain: 50<br>Gray Map: E<br>Depth: 12cm<br>Focus Points: 1<br>Dynamic Range: 69
Example ultrasound image and measurement of the gluteus medius.	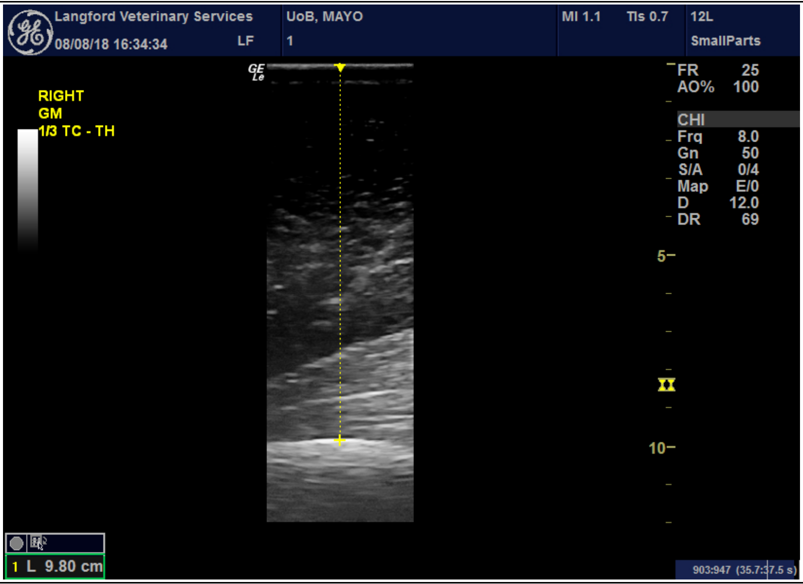
Photograph to demonstrate ultrasound transducer placement to image the gluteus medius muscle.	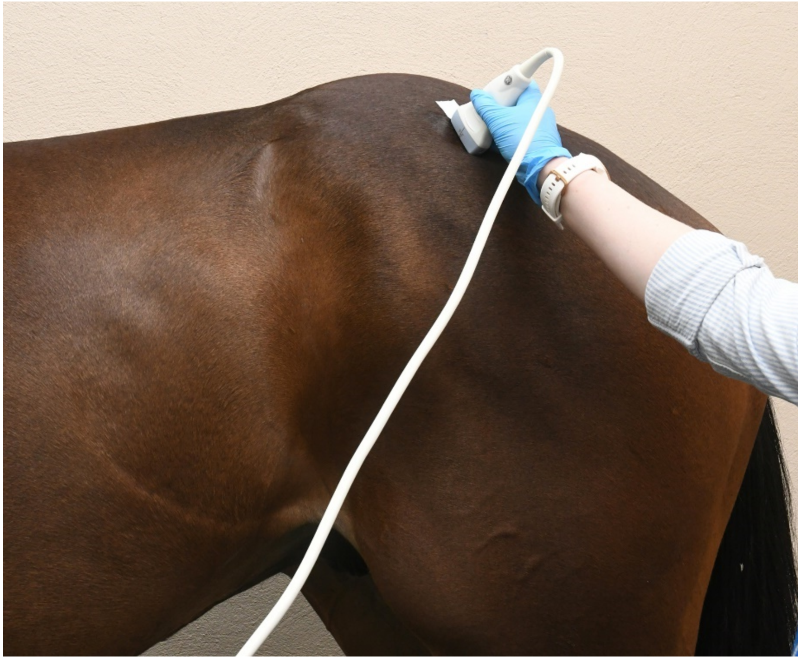

VASTUS LATERALIS

**Description**	Horse standing square behind. Place a marker over the femur in line with the junction between the flank and thigh. Position the transducer transverse to the femur, with minimal pressure.
**Image Measurement**	Measure from the skin to the surface of the femur
**Ultrasound Machine Settings**	Transducer: Linear<br>Frequency: 13MHz<br>CHI: on<br>Gain: 46<br>Gray Map: F<br>Depth: 10cm<br>Focus Points: 1<br>Dynamic Range: 69
Example ultrasound image and measurement of the vastus lateralis.	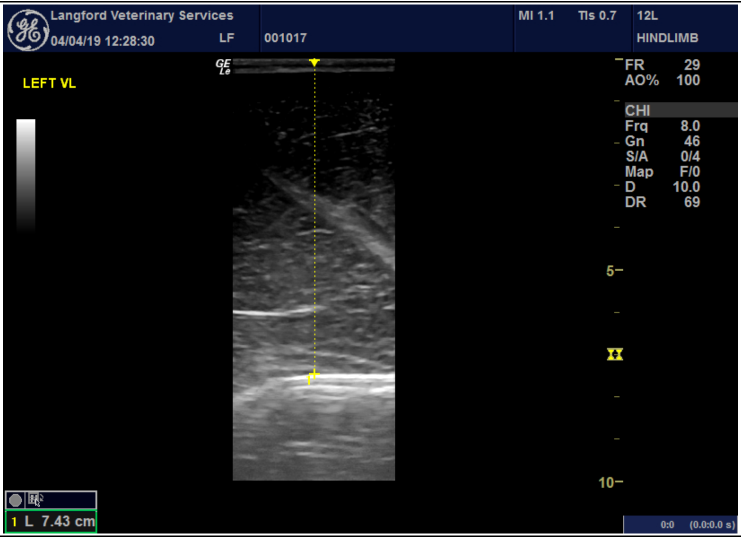
Photograph to demonstrate ultrasound transducer placement for the vastus lateralis muscle. A marker is placed over the femur, on a line between the junction of the thigh and flank, to guide ultrasound transducer placement.	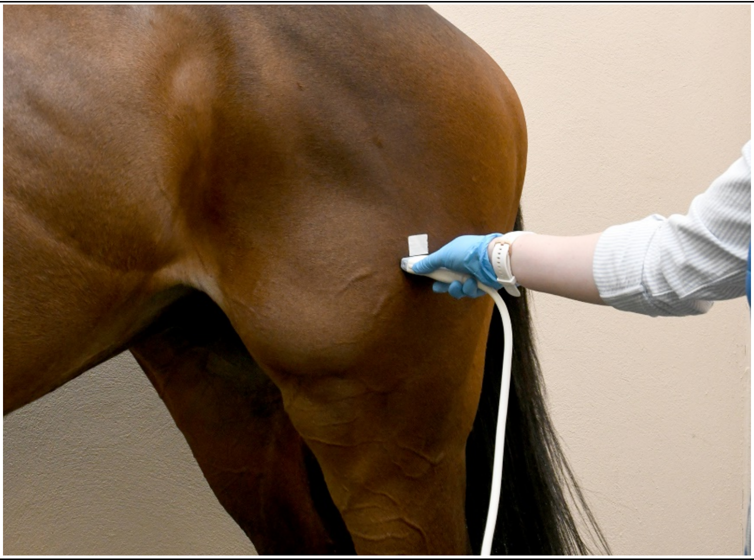
