# Supplementary material for: Training the equine respiratory muscles: Ultrasonographic measurement of muscle size
Source: Equine Vet J. 2022 Jun 19;55(2):295–305. doi: 10.1111/evj.13598 (PMC10084327; doi:10.1111/evj.13598)
Supplement: Supplementary file 3 — Table S1 Inspiratory muscle training schedule. [file EVJ-55-295-s004.pdf]

Equine Veterinary Journal BEVA

Table S1: Inspiratory muscle training schedule.

IMT – High Load

Phase 1: Intersurgical® Valves

Day	Pressure (cmH_2O) (Session 1)	Pressure (cmH_2O) Session 2
1	5	5
2	5	10
3	10	10
4	10	10
5	10	12.5
6	10	12.5
7	12.5	12.5
8	12.5	15
9	12.5	15
10	15	15
11	15	15
12	15	20
13	15	20
14	20	20
15	20	20

Phase 2: POWERbreathe® valve

Day	Pressure (cmH_2O) (Session 1)	Pressure (cmH_2O) Session 2
16	20	20
17	20	20
18	20	22.5
19	20	22.5
20	22.5	22.5
21	22.5	22.5
22	22.5	25
23	22.5	25
24	25	25
25	25	25
26	25	27.5
27	25	27.5
28	27.5	27.5
29	27.5	27.5
30	27.5	30
31	27.5	30
32	30	30
33	30	30
34	30	32.5
35	30	32.5
36	32.5	32.5
37	32.5	32.5
38	32.5	35
39	32.5	35
40	35	35
41	35	35
42	35	37.5
43	35	37.5
44	37.5	37.5
45	37.5	37.5
46	37.5	4
47	37.5	4
48	4	4
49	4	4
50	4	42.5

IMT – Low Load

Day	Resistance (Session 1)	Resistance (Session 2)	Extra (5 breaths)
1	2.5	2.5	
2	2.5	2.5	
3	2.5	2.5	
4	2.5	2.5	
5	2.5	2.5	10
6	2.5	2.5	
7	2.5	2.5	
8	2.5	2.5	
9	2.5	2.5	
10	2.5	2.5	12.5
11	2.5	2.5	
12	2.5	2.5	
13	2.5	2.5	
14	2.5	2.5	
15	2.5	2.5	15
16	2.5	2.5	
17	2.5	2.5	
18	2.5	2.5	
19	2.5	2.5	
20	2.5	2.5	20
21	2.5	2.5	
22	2.5	2.5	
23	2.5	2.5	
24	2.5	2.5	
25	2.5	2.5	20

Day	Resistance (Session 1)	Resistance (Session 2)	Extra (5 breaths)
26	2.5	2.5	
27	2.5	2.5	
28	2.5	2.5	
29	2.5	2.5	
30	2.5	2.5	20
31	2.5	2.5	
32	2.5	2.5	
33	2.5	2.5	
34	2.5	2.5	
35	2.5	2.5	20
36	2.5	2.5	
37	2.5	2.5	
38	2.5	2.5	
39	2.5	2.5	
40	2.5	2.5	20
41	2.5	2.5	
42	2.5	2.5	
43	2.5	2.5	
44	2.5	2.5	
45	2.5	2.5	20
46	2.5	2.5	
47	2.5	2.5	
48	2.5	2.5	
49	2.5	2.5	
50	2.5	2.5	20
