# Supplementary material for: Training the equine respiratory muscles: Ultrasonographic measurement of muscle size
Source: Equine Vet J. 2022 Jun 19;55(2):295–305. doi: 10.1111/evj.13598 (PMC10084327; doi:10.1111/evj.13598)
Supplement: Supplementary file 4 — Table S2 Results from the repeated measures model comparing the muscle size measurements at each timepoint, for the measurements obtained from yard 1 and yard 2. [file EVJ-55-295-s006.pdf]

Equine Veterinary Journal BEVA

Table S2: Results from the repeated measures model comparing the muscle size measurements at each timepoint, for the measurements obtained from yard 1 and yard 2. The B values are reported where a significant difference was obtained. A positive B value is indicative of an increase in size measurement; a negative B value is indicative of a decrease in size measurement.

Variable	Overall P value	Time point A vs B (B [95% CI]) (cm/12weeks)	Time point A vs C (B [95% CI]) (cm/22-24weeks)	Time point B vs C (B [95% CI]) (cm/10-12weeks)
**Left TH**	*<0.001*	*0.071*	*<0.001*<br>0.03 [0.02 to 0.04]	*0.003*<br>0.02 [0.01 to 0.03]
**Right TH**	*<0.001*	*0.532*	*<0.001*<br>0.03 [0.02 to 0.05]	*<0.001*<br>0.03 [0.02 to 0.04]
**Left CT**	*0.001*	*0.876*	*0.001*<br>0.07 [0.03 to 0.11]	*0.001*<br>0.07 [0.03 to 0.11]
**Right CT**	*0.015*	*0.961*	*0.011*<br>0.05 [0.01 to 0.09]	*0.010*<br>0.05 [0.01 to 0.08]
**Left GH**	*0.001*	*<0.001*<br>0.11 [0.05 to 0.17]	*0.085*	*0.077*
**Right GH**	*0.019*	*0.034*<br>0.07 [0.01 to 0.13]	*0.998*	*0.020*<br>-0.07 [-0.14 to -0.01]
**Left GG**	*0.053*	N/A	N/A	N/A
**Right GG**	*0.016*	*0.333*	*0.005*<br>-0.16 [-0.27 to -0.05]	*0.044*<br>-0.10 [-0.21 to 0.00]
**Lingual process**	*0.149*	N/A	N/A	N/A
**STH**	*<0.001*	*0.003*<br>0.23 [0.08 to 0.38]	*<0.001*<br>0.03 [0.02 to 0.04]	*0.293*
**Left ECR**	*<0.001*	*0.262*	*<0.001*<br>0.24 [0.14 to 0.34]	*<0.001*<br>0.18 [0.09 to 0.28]
**Right ECR**	*0.108*	N/A	N/A	N/A
**Left GM**	*<0.001*	*<0.001*<br>0.32 [0.15 to 0.48]	*0.003*<br>0.29 [0.10 to 0.48]	*0.592*
**Right GM**	*<0.001*	*<0.001*<br>0.30 [0.14 to 0.45]	*<0.001*<br>0.57 [0.39 to 0.75]	*0.002*<br>0.27 [0.10 to 0.43]
**Left VL**	*0.737*	N/A	N/A	N/A
**Right VL**	*0.041*	*0.276*	*0.186*	*0.012*<br>-0.14 [-0.25 to -0.03]
**Left D Insp**	*<0.001*	*<0.001*<br>0.17 [0.12 to 0.21]	*0.004*<br>0.08 [0.03 to 0.13]	*0.001*<br>-0.09 [-0.14 to -0.04]
**Left D Exp**	*<0.001*	*<0.001*<br>0.09 [0.05 to 0.12]	*0.251*	*<0.001*<br>-0.11 [-0.14 to -0.07]
**Right D Insp**	*0.007*	*0.013*<br>0.05 [0.01 to 0.09]	*0.713*	*0.005*<br>-0.06 [-0.10 to -0.02]
**Right D Exp**	*0.002*	*0.456*	*0.013*<br>-0.04 [-0.08 to -0.01]	*0.001*<br>-0.06 [-0.09 to -0.02]

TH: thyrohyoideus; CT: cricothyroideus; GH: geniohyoideus; GG: genioglossus; STH: sternothyrohyoideus; ECR: extensor carpi radialis; GM: gluteus medius; VL: vastus lateralis; D: diaphragm; Insp: inspiration; Exp: expiration.
