# Supplementary material for: Training the equine respiratory muscles: Ultrasonographic measurement of muscle size
Source: Equine Vet J. 2022 Jun 19;55(2):295–305. doi: 10.1111/evj.13598 (PMC10084327; doi:10.1111/evj.13598)
Supplement: Supplementary file 5 — Table S3 Results from the univariate model, adjusted for timepoint, to assess the effect of inspiratory muscle training on the ultrasound size measurements. [file EVJ-55-295-s002.pdf]

Equine Veterinary Journal BEVA

Table S3: Results from the univariate model, adjusted for timepoint, to assess the effect of inspiratory muscle training on the ultrasound size measurements. Only the significant results are displayed. A positive B value is indicative of an increase in size measurement; a negative B value is indicative of a decrease in size measurement.

**Variable**		**P (between groups)**	**Unit change in variable per timepoint (B) [95% CI] (cm/22-24weeks)**	**SE**	**P (within group)**
**STH**	IMT (High Load)<br>IMT (Low Load)	0.024	0.24 [0.11 to 0.37]<br>0.00 [-0.16 to 0.16]	0.06<br>0.08	0.001<br>0.985
**Left Diaphragm Insp**	IMT (High Load)<br>IMT (Low Load)	0.011	0.11 [0.05 to 0.17]<br>- 0.01 [-0.08 to 0.06]	0.03<br>0.04	<0.001<br>0.770
**Left Diaphragm Exp**	IMT (High Load)<br>IMT (Low Load)	0.012	0.03 [-0.10 to 0.08]<br>-0.06 [-0.11 to -0.00]	0.02<br>0.03	0.127<br>0.040
**Right Diaphragm Insp**	IMT (High Load)<br>IMT (Low Load)	0.003	0.02 [-0.02 to 0.06]<br>-0.07 [-0.12 to -0.03]	0.02<br>0.02	0.335<br>0.002
**Right Diaphragm Exp**	IMT (High Load)<br>IMT (Low Load)	0.007	-0.01 [-0.04 to 0.02]<br>-0.07 [-0.11 to -0.04]	0.01<br>0.02	0.731<br><0.001

STH: Sternothyrohyoideus; IMT: Inspiratory Muscle Training; Insp: Inspiration; Exp: Expiration
