# Supplementary material for: Training the equine respiratory muscles: Ultrasonographic measurement of muscle size
Source: Equine Vet J. 2022 Jun 19;55(2):295–305. doi: 10.1111/evj.13598 (PMC10084327; doi:10.1111/evj.13598)
Supplement: Supplementary file 6 — Table S4 Results from the univariate model, adjusted for timepoint, to investigate the effect of yard on the ultrasound size measurements. [file EVJ-55-295-s003.pdf]

Equine Veterinary Journal BEVA

Table S4: Results from the univariate model, adjusted for timepoint, to investigate the effect of yard on the ultrasound size measurements. Only the significant results are displayed. A positive B value is indicative of an increase in size measurement; a negative B value is indicative of a decrease in size measurement.

Variable		P (between groups)	Unit change in variable per timepoint (B) [95% CI] (cm/22-24weeks)	SE	P (within group)
**Left TH**		0.041			
	Yard (1)		0.02 [0.01 to 0.03]	0.01	<0.001
	Yard (2)		0.01 [0.00 to 0.02]	0.00	0.038
**Left GH**		0.003			
	Yard (1)		-0.03 [-0.08 to 0.02]	0.03	0.269
	Yard (2)		0.08 [0.03 to 0.12]	0.02	0.770
**Right GH**		<0.001			
	Yard (1)		-0.08 [-0.13 to -0.03]	0.03	0.003
	Yard (2)		0.06 [0.02 to 0.10]	0.02	0.008
**Lingual process**		0.029			
	Yard (1)		-0.05 [-0.10 to -0.01]	0.02	0.013
	Yard (2)		0.01 [-0.03 to 0.04]	0.02	0.677
**Left ECR**		0.030			
	Yard (1)		0.19 [0.11 to 0.26]	0.04	<0.001
	Yard (2)		0.07 [0.01 to 0.14]	0.03	0.031
**Left GM**		0.045			
	Yard (1)		0.03 [-0.12 to 0.18]	0.08	0.661
	Yard (2)		0.23 [0.11 to 0.36]	0.06	<0.001
**Right GM**		0.017			
	Yard (1)		0.16 [0.03 to 0.30]	0.07	0.018
	Yard (2)		0.39 [0.26 to 0.49]	0.06	<0.001

TH: thyrohyoideus; GH: geniohyoideus; ECR: extensor carpi radialis; GM: gluteus medius; SE: Standard Error.
