# Supplementary material for: Training the equine respiratory muscles: Ultrasonographic measurement of muscle size
Source: Equine Vet J. 2022 Jun 19;55(2):295–305. doi: 10.1111/evj.13598 (PMC10084327; doi:10.1111/evj.13598)
Supplement: Supplementary file 7 — Table S5 Results from the univariate model, adjusted for timepoint, to assess the effect of airway surgery on the ultrasound size measurements. [file EVJ-55-295-s005.pdf]

Equine Veterinary Journal BEVA

Table S5: Results from the univariate model, adjusted for timepoint, to assess the effect of airway surgery on the ultrasound size measurements. Only the significant results are displayed. A positive B value is indicative of an increase in size measurement; a negative B value is indicative of a decrease in size measurement.

**Variable**		**P (between groups)**	**Unit change in variable per timepoint (B) [95% CI] (cm/22-24weeks)**	**SE**	**P (within group)**
**Left GM**		0.030			
	Airway Surgery (yes)		0.07 [-0.05 to 0.19]	0.06	0.273
	Airway Surgery (no)		0.29 [0.13 to 0.35]	0.08	<0.001
**Right GM**		0.037			
	Airway Surgery (yes)		0.21 [0.10 to 0.33]	0.06	<0.001
	Airway Surgery (no)		0.41 [0.26 to 0.55]	0.07	<0.001

GM: gluteus medius.
